# “From the technology came the idea”: safe implementation and operation of a high quality teleradiology model increasing access to timely breast cancer assessment services for women in rural Australia

**DOI:** 10.1186/s12913-020-05922-y

**Published:** 2020-11-30

**Authors:** Karen Johnston, Deborah Smith, Robyn Preston, Rebecca Evans, Karen Carlisle, Janet Lengren, Helen Naess, Elizabeth Phillips, Greg Shephard, Lorraine Lydiard, Debbie Lattimore, Sarah Larkins

**Affiliations:** 1 College of Medicine and Dentistry, James Cook University, QLD, Douglas, 4814 Australia; 2 School of Health, Medical and Applied Sciences, CQUniversity, QLD, Townsville, 4810 Australia; 3 BreastScreen Queensland, PO Box 2368, Fortitude Valley BC, Qld 4006 Australia; 4 BreastScreenNT, Casuarina, NT 0810 Australia; 5 BreastScreen NSW, Alexandria, NSW 1435 Australia

**Keywords:** Telemedicine, Telehealth, Teleradiology, Telesonography, Telemammography, Breast cancer, Diagnosis, Primary health care, Rural and remote, Innovation

## Abstract

**Abstract:**

Breast cancer is the most commonly diagnosed cancer in Australian women. Providing timely diagnostic assessment services for screen-detected abnormalities is a core quality indicator of the population-based screening program provided by BreastScreen Australia. However, a shortage of local and locum radiologists with availability and appropriate experience in breast work to attend onsite assessment clinics, limits capacity of services to offer assessment appointments to women in some regional centres. In response to identified need, local service staff developed the remote radiology assessment model for service delivery. This study investigated important factors for establishing the model, the challenges and enablers of successful implementation and operation of the model, and factors important in the provision of a model considered safe and acceptable by service providers.

**Methods:**

Semi-structured interviews were conducted with service providers at four assessment services, across three jurisdictions in Australia. Service providers involved in implementation and operation of the model at the service and jurisdictional level were invited to participate. A social constructivist approach informed the analysis. Deductive analysis was initially undertaken, using the interview questions as a classifying framework. Subsequently, inductive thematic analysis was employed by the research team. Together, the coding team aggregated the codes into overarching themes.

**Results:**

55 service providers participated in interviews. Consistently reported enablers for the safe implementation and operation of a remote radiology assessment clinic included: clinical governance support; ability to adapt; strong teamwork, trust and communication; and, adequate technical support and equipment. Challenges mostly related to technology and internet (speed/bandwidth), and maintenance of relationships within the group.

**Conclusions:**

Understanding the key factors for supporting innovation, and implementing new and safe models of service delivery that incorporate telemedicine, will become increasingly important as technology evolves and becomes more accessible. It is possible to take proposed telemedicine solutions initiated by frontline workers and operationalise them safely and successfully: (i) through strong collaborative relationships that are inclusive of key experts; (ii) with clear guidance from overarching bodies with some flexibility for adapting to local contexts; (iii) through establishment of robust teamwork, trust and communication; and, (iv) with appropriate equipment and technical support.

**Supplementary Information:**

The online version contains supplementary material available at 10.1186/s12913-020-05922-y.

## Background

Breast cancer is the most commonly diagnosed cancer in Australian women [1]. To enhance early detection and treatment, all women in Australia aged between 50 and 74 years are invited to attend a screening mammogram every 2 years at their nearest BreastScreen location or mobile outreach service. According to the latest publicly available data, 54.5% of Australian women in the target age group participate in this screening (1.8 million women in 2016–17; 2). This is considerably lower for Aboriginal and Torres Strait Islander women at 40.7%, and culturally and linguistically diverse women at 45.8% in 2016–17 [2]. Approximately 11% of women attending their first screening and 3.5% of women attending subsequent screens are recalled for further diagnostic investigation at an assessment clinic [2].

Breast screening and diagnostic assessment are both vital to the early detection of breast cancer. However, many issues impact on the appropriate and acceptable provision of these services in non-metropolitan areas. Such challenges include: workforce maldistribution (in a geographic sense and by specialty) [3, 4]; populations dispersed over large geographic areas outside metropolitan centres; current health system constraints (e.g. focus on sustainability and cost containment); and, community expectations for timely and acceptable care [5]. These challenges make it particularly difficult to provide care for non-metropolitan communities and can reinforce persisting health inequities seen amongst rural, remote, and Aboriginal and Torres Strait Islander populations [6–9]. A number of health service and system innovations seek to facilitate sustainable provision of health services in rural and remote areas, including the use of telemedicine technologies.

Telemedicine is widely used in a variety of diagnostic, therapeutic and educational settings to: i) increase the access of rural and remote residents to specialist health care and expertise; ii) overcome workforce shortages in regional, rural and remote areas through remote service provision [10–13]; or iii) provide supervision or support to rural and remote health professionals and trainees [14]. The use of teleradiology for diagnostic imaging studies has been widely adopted in Australia and in other similarly resourced countries, following the establishment of digital imaging [15]. Teleradiology is defined as “...the electronic transmission of diagnostic radiological images in digital form between locations (acquisition site to reporting site) for diagnosis and reporting by a clinical radiologist or any other appropriately credentialed medical specialist using a bi-directional data communication link that keeps all patient data secure” ( [16] , p.56).

Providing timely diagnostic assessment services for screen-detected abnormalities is a core quality indicator for BreastScreen Australia services [17]. However, a shortage of local and locum radiologists with availability and appropriate experience in breast work to attend onsite assessment clinics, limits capacity of services to offer assessment appointments to women in some regional centres. Encouraged by the capabilities of Picture Archiving and Communication Systems (PACS), introduced for routine use in BreastScreen services, a team of BreastScreen service staff located in a regional centre developed a novel model of radiology service delivery in assessment clinics: the remote radiology assessment model. After a period of implementation and testing at a pilot site, the remote radiology assessment model was further implemented with support and under protocol requirements of the BreastScreen Australia National Quality Management Committee (BSANQMC) at seven BreastScreen assessment clinics, across three jurisdictions (States and Territories) in Australia.

### Remote radiology assessment model for service delivery

Under the remote radiology assessment model, radiologists participate in assessment clinics from a location that is remote to the site, through appropriate technology and telehealth facilities. There are slight variations in how the model operates at different service locations, depending on factors such as team preferences for service flow, and professional characteristics and skill-sets of onsite staff. However, the availability of synchronous telesonography (transmission of ultrasound to a remote location using telehealth technology) is a core feature.

**Fig. 1:**
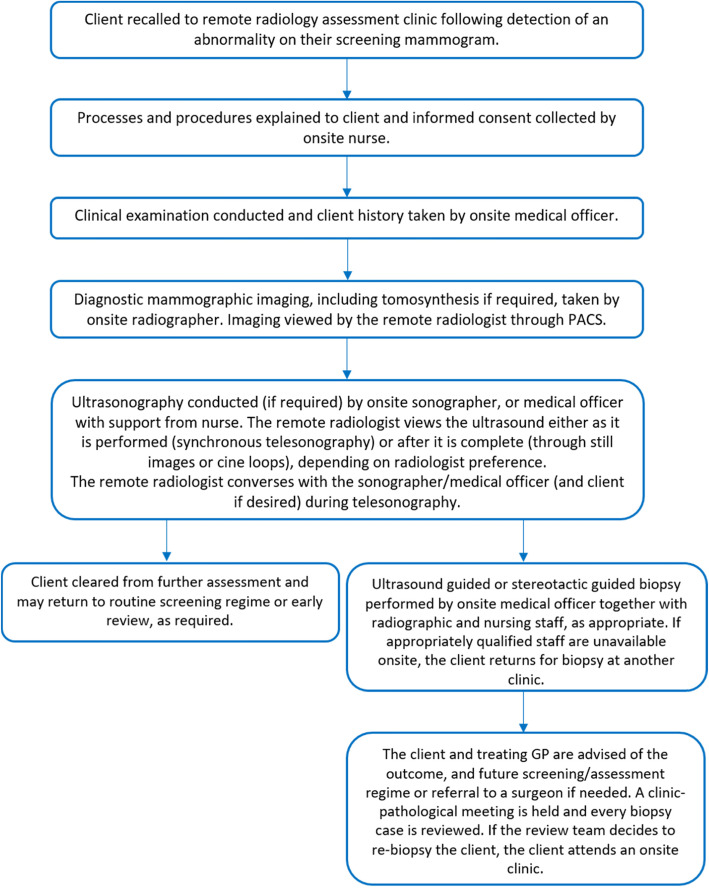
The remote radiology assessment model for service delivery

In a typical onsite assessment clinic, clients move through the following processes: consent; clinical examination and history; diagnostic mammography; ultrasonography (as required); biopsy (as required); and a concluding consultation with a medical officer or nurse. The clinical assessment team, consisting of a radiologist, nurses, doctors, sonographers and radiographers, are all located onsite. The radiologist reports on imaging from the same location as the assessment team and client. In a remote radiology assessment clinic, clients move through this same process, however the radiologist views and reports on imaging from a remote location (Fig. 1). Communication between onsite staff and the remote radiologist occurs via telehealth technology throughout the clinic, and before and after the clinic, as needed. This communication may occur through regular team meetings at certain intervals during the clinic, and/or between or during client procedures. Clients may also speak with the remote radiologist using telehealth technology if required.

The remote radiology assessment model uses teleradiology for diagnostic assessment of mammographic images by a remote radiologist. The model also incorporates teleradiology in the transmission of client ultrasound imaging captured onsite to the remote location (telesonography). Transmission may be asynchronous (otherwise known as store-and-forward) or synchronous (in real-time). Ultrasound still images and/or cine loops are sent through the PACS to the remote radiologist for review and diagnostic reporting (asynchronous telesonography). If the radiologist requests, synchronous telesonography is enabled. Synchronous telesonography uses telehealth technology to facilitate real-time viewing and guidance of an ultrasound procedure by the remote radiologist, where the ultrasound and any procedures are conducted by the onsite sonographer or appropriately skilled medical officer. The use of synchronous telesonography during client assessment depends on the preferences of the remote radiologist, with some preferring to use the technology for every assessment, and others using it if needed to inform their decision making.

An onsite medical officer with appropriate skills and experience conducts ultrasound-guided or stereotactic-guided biopsies together with radiographic and nursing staff. If appropriately qualified staff are unavailable onsite, the client returns for biopsy at another assessment clinic when a radiologist is onsite. All client cases involving a biopsy procedure are reviewed at a clinico-pathological meeting (involving at minimum a radiologist, medical officer, pathologist) after the clinic. In the case that the team decides to re-biopsy a client, the client attends an onsite clinic.

Understanding the key factors for supporting innovation, and implementing new and safe models of service delivery that incorporate telemedicine, will become increasingly important as technology evolves and becomes more accessible. Further, an in-depth understanding of the model, implementation and outcomes for health professionals is important for informing any potential implementation of the model at other assessment services. This paper outlines the qualitative findings of a study evaluating the implementation of the remote radiology assessment model at four BreastScreen services across three Australian jurisdictions. The qualitative component of the study aimed to identify important factors for establishing the remote radiology assessment model, challenges and enablers of successful implementation and operation of the model, and important factors in a model considered safe and acceptable by service providers. Other aspects of the remote radiology assessment model were investigated as part of the overall evaluation study including client perceptions of the model, and a quantitative analysis of quality and safety using clinical outcomes. These findings are presented elsewhere (Smith D, et al. Client perceptions of BreastScreen remote radiology assessments, unpublished) [18, 19].

## Methods

Drawing on a social constructivist perspective [20], this qualitative study sought to understand participants’ subjective experience of the phenomena under study. By adopting this approach, researchers acknowledge that meanings of these experiences are formed through interaction with historical, social and cultural contexts whilst recognising their own background shapes their interpretation [21].

### Sites

Four BreastScreen services delivering remote radiology assessment services in Australia participated in the study. Two services were situated in outer regional Australia (Remoteness Area (RA) 3; RA being a measure of relative accessibility and remoteness), one service was located in inner regional Australia (RA2), and one service was located in metropolitan Australia (RA1 [22];). At all four sites, the remote radiology assessment model was implemented in response to a chronic shortage of local and locum radiologists, with availability and appropriate experience in breast work, to attend assessment clinics. Service catchment populations were mostly spread over large geographic areas with clients often traveling considerable distances from rural and remote areas to attend the assessment service.

### Participants and recruitment

Prior to commencement of the study, service providers were made aware, through BreastScreen communications, that an evaluation of the remote radiology assessment model was being conducted by an independent research organisation. Service providers at each participating BreastScreen service, and other service providers that coordinated and supported service delivery across the three jurisdictions, were invited by the research team to participate in semi-structured interviews. Participants were recruited through site visits, email and snowball recruitment. BreastScreen service providers included medical officers, breast nurses, remote radiologists, radiographers, sonographers, data managers, data administrators, health promotion officers and managers.

Service providers who were external to BreastScreen and provided services that enabled or supported the remote radiology assessment model were invited to participate in semi-structured interviews using the same recruitment process described above. Such providers included telehealth or information technology service specialists. Service providers who had worked with clinics and coordinating bodies previously, or assisted in the implementation of the model, were also invited to participate in a semi-structured interview.

### Data collection

Semi-structured interviews offered flexibility to explore concepts as they emerged from interview participants, and were therefore the most appropriate data collection tool to address the aims of this study [23]. An interview guide was developed based on a review of the teleradiology literature and aims of the study (Additional File 1). The guide was piloted within the research team and minor changes were made accordingly. The interview guide aimed to elicit views on the remote radiology assessment model, particularly:

(i) Aspects of the remote radiology model that have benefits for staff and/or clients and service operation;
(ii) Aspects of the remote radiology model that could pose risks to clients and/or staff or limitations to the service provided;
(iii) Processes that worked well or posed challenges in the smooth implementation of the remote radiology model at their site;
(iv) What could be done differently to improve the remote radiology model at their site;
(v) What advice they would have for other sites seeking to implement the remote radiology model; and
(vi) Staff perceptions of client acceptance and team functioning that affect the safety and quality of the care provided via the remote radiology model.

Interviews with health service providers were conducted by two authors, both female research officers with several years of experience in conducting qualitative research (KJ; PhD candidate, and DS; PhD). Informed consent to participate was collected. Interviews were audio-recorded, with consent, and transcribed verbatim. Field notes were made after interviews. Transcripts were imported into the qualitative management software package, QSR NVivo (version 12 [24];), to facilitate data management and qualitative analysis by the research team. Transcripts were offered to participants for comment however few took up this option. One participant provided further information to add to their transcript.

### Analysis

Deductive analysis was initially undertaken, using the interview questions as a classifying framework. Subsequently, inductive thematic analysis was employed to allow for the construction of themes directly from the data [25]. Each transcript was independently coded by two members of the research team. The team met on several occasions to discuss the codes and themes derived from this analysis. Variations between services and between types of service providers were considered. Differences between team members’ coding were resolved by consensus, and involved the team returning to the transcripts to consider and verify the context of the differences. Together, the coding team aggregated the codes into overarching themes which are presented in the results section.

Various strategies were used throughout the analysis process to enhance rigour and trustworthiness of findings. Regular meetings to discuss interpretation of codes and themes, sharing of memos and notes, co-coding of qualitative data, data triangulation (using multiple data collection methods and sources including interviews with a range of service providers, documents and field notes) and consideration of disparate views ensured balanced investigation of service provider perspectives. Provision of ample and rich quotes from participants enhanced the connection between data and conclusions.

## Results

A total of 55 service providers participated in interviews between November 2017 and May 2019 (Site 1, *n* = 13; Site 2, *n* = 8; Site 3, *n* = 14; Site 4, *n* = 9; coordinating and supporting service providers, *n* = 11). Interviews were conducted face to face in private offices at clinics or via telephone. Service staff participants were medical officers, breast physicians, nurses, radiologists, radiographers, sonographers, service data managers, administrators, receptionists, service managers and a health promotion officer. Coordinating and supporting service providers across the three jurisdictions included managers, project officers, data specialists, and telehealth and medical physics/biomedical specialists (Table 1). Three interviews were conducted with multiple participants at the same time (group interviews). Interviews ranged from 6 to 54 min in duration with an average duration of 22 min.

**Table 1:** Number of participants and their roles

Participant role	Number of participants
Medical officer/Breast physician^a^	8
Clinical nurse/Nurse counsellor/Breast nurse	7
Radiologist	8
Radiographer/Sonographer	8
Service data manager/Service manager	3
Data administrator	3
Receptionist/Administrator	5
Health promotion officer	1
Coordinating and supporting service providers	
- Managers	5
- Project officers	2
- Data specialists	2
- Telehealth, medical physics/biomedical specialists	3

^a^To maintain anonymity in reporting of qualitative findings, breast physicians are referred to as medical officers throughout the report, noting that the term ‘breast physician’ refers to a different role than ‘medical officer’ in the context of some remote radiology assessment models. Likewise, there were various titles for nurses reflecting the diverse roles and specialty areas for nurses involved in assessment clinics. To maintain anonymity in reporting, the term ‘nurse’ is used

For the majority of participants, remote radiology assessment clinics represented their first experience of a service model that was based on teleradiology. Those participants with previous experience of teleradiology were mostly professionals in the field of medical imaging. Interviewees had been in their current role for a variety of time periods, with one site having a very experienced team with more than 5 years in the current role for most staff members. At another site the majority of participants had less than 1 year to 5 years’ experience in their current role, and the other two sites had a range of experience in between.

Overall, service providers were satisfied with the remote radiology assessment model. Participants reported that it was a valuable model for providing high quality and timely assessment services to women living in regional, rural and remote areas of the country. There was a clear indication throughout interviews that service providers felt the remote radiology assessment model improved availability of assessment clinics to clients, improved service timeliness measures, was functional in terms of clinic processes, and increased communication and teamwork. It was clear that service providers were passionate about providing the best quality care and client experience possible, and were willing to put in the extra time and effort required to run clinics using the remote radiology assessment model (client views reported elsewhere (Smith D, et al. Client perceptions of BreastScreen remote radiology assessments, unpublished) [19];).

The reported enablers and challenges for the safe implementation and operation of a remote radiology assessment clinic were categorised into the following themes: clinical governance support; ability to adapt; strong teamwork, trust and communication; and, adequate technical support and equipment. Each of these themes are discussed below.

### Challenges and enablers of safe implementation and operation of the remote radiology model

#### Clinical governance support

Engaging key people in a collaborative approach was a valuable enabler for initial implementation of the model. Key people included technology experts from partner organisations, jurisdictional management and clinical governance staff, service clinicians and project officers. Involvement of telehealth experts, biomedical equipment experts, and medical physicists ensured that the technology and processes worked at optimal quality to facilitate safety in the remote radiology model. Collaboration between key people occurred through the formation of a governance structure and advisory committee at jurisdictional level and regular operational team meetings at the local level.

The model was implemented across the jurisdictions in accordance with protocol requirements of the BSANQMC. Importantly, the overarching protocol requirements allowed jurisdictions flexibility in adapting the model to their local context. However, it was noted by some participants that clearer guidance from the national committee was desired to overcome different interpretations of the protocol guidelines, for instance guidelines for the relative frequency of onsite versus remote radiology clinics. Governance bodies in each jurisdiction developed their own general procedures and standardisation of paperwork, in keeping with their local operational and service flows. Standardised processes to monitor the clinical outcomes of the model were put in place at the jurisdictional level.

Participants reported that services already conducting optimal onsite assessment clinics could consider using the remote radiology model. Ensuring that remote radiology assessment clinics were staffed with appropriately skilled and experienced staff was identified as fundamental for the success of the model and a few participants noted that this could be a risk to the sustainability of the model. In addition, some participants perceived professional risks associated with an expanded scope of duty, as described by a medical officer (site 1), *“The buck stops with me. I’m the one that gets the blame if there’s a complication. I have to wear that risk.”* Some participants perceived that this may affect future recruitment of health professionals to be involved in the remote radiology model.

#### Ability to adapt

Participants reported a need to adapt service and administrative processes for delivery of remote radiology assessment clinics. This required considerable effort prior to operationalisation. Throughout implementation, continually reviewing processes and troubleshooting problems as they emerged enabled the smooth operation of the model. For example, across sites, approaches for communication between onsite service providers and the remote radiologist continued to develop as service providers reflected on, and improved, ways of sharing and discussing client information.

*“You need to think outside the box. You can’t be hamstrung by ‘we’ve always done it this way’. You’ve gotta … roll with the punches … a [remote] radiologist who’s going, ‘but I can’t see what you’re talking about’ … you have to think, ‘how can I communicate this?’ ‘I know, put an arrow on it’.”*

*–* medical officer (site 1).

Adapting to service delivery using the model, with changed processes and the use of technology, was challenging for some participants initially. For the model to operate well, a highly organised approach to the clinic was needed, with all members of the team aware of their role and able to perform their work to a high standard. Team acceptance and team investment in making the remote radiology model work may have promoted adaptation to delivery of the model, as described by a medical officer (site 2) “*… when you are very invested in something, the steps that have to be taken don’t seem like challenges”*.

Participants commonly reported that staff recognised the need for, and were supportive of the model, and were *“more than happy to change their process, to have the possibility of a remote clinic”* (manager; site 4). Time for participants to adapt to the different work flow and team dynamics was essential, as described by a radiographer (site 4), *“I think initially when people are getting used to it, it [team functioning] obviously does change because you are getting used to that different dynamic of how do you communicate and things like that. But then as we’ve adapted to that and … now, it’s kind of just normal.”*

#### Teamwork, trust and communication

Well-established, strong working relationships, driven by the importance of trust and good communication amongst the team was identified as a significant theme in implementation processes. Ultimately, onsite and remote providers needed to have working relationships that meant they could *“… discuss and question things without offence”* (remote radiologist, site 3). As one medical officer (site 2) described when asked about how ‘knowing’ the remote radiologist was important, *“I think it just seems more friendly, you sort of feel like, if you did disagree or if you had an extra question, you feel like you can just butt in like you would if they were standing in front of you. Whereas if you have never met them before, if you had real doubts, you would probably still butt in I guess, but … it’s just easier … know [ing] who [is] on the other end of the phone.”*

It was important that there were opportunities to develop and maintain strong team relationships when implementing the remote radiology model, *“so that … we all know each other and know each other well enough so that you can be concentrating on paying attention and have your protocols in place”* (medical officer; site 4). Many participants believed that strong relationships between service providers could be formed through a long history of working together. At one site, consistency of staff at onsite and remote radiology assessment clinics was facilitated through a contractual arrangement. In this case, existing relationships were built upon and maintained through regular conduct of assessment clinics. For other sites, conducting an onsite assessment clinic at least once a year with the radiologist who worked in the remote radiology model, was reported to be acceptable for maintaining strong team relationships.

It was noted by a medical officer (site 1) that trust was implicit in medicine: *“… that’s basically medicine, a lot of it [is] just on trust, you … trust this person to consent this person fully so they understand what they are doing. I trust this person to do an ultrasound and do it well so they are actually imaging the area they are supposed to image. I trust the radiographers to take the right images and not to take five or six before they produce one.”* However, it was commonly reported that the telehealth aspect of remote radiology assessment clinics required an extra degree of trust. Trust involved confidence in the skills of all clinical service providers, a knowledge about how each team member worked, and their work capabilities.

*“… that relationship that you build up by working with somebody over a long period of time, is absolutely imperative with this process. The other thing with it, is there’s no position for any weak links when you are doing it remotely. You’ve got to have faith in every single member of the team, so even if there is a locum in town … you’ve got to have met them, worked with them”.*

– remote radiologist (site 1).

For remote radiologists, comfort with reporting in the remote radiology assessment model was strongly linked with their confidence and trust in onsite clinical service providers. Trust in onsite professionals in conducting ultrasound and performing biopsies was of particular importance, as emphasised by participants:

*“I know the sonographers that are there and I have confidence in them, so I’m happy to look at their … images and I guess I’m less, much less, concerned because I know the sonographer there. And that comes with time, you know, knowing how good the sonographer is.”*

*–* remote radiologist (site 4).

*“… you’ve got to trust the people you are working with. The radiologists have got to trust me. I’ve got to trust them. They’ve got to trust my ability to do biopsies. They’ve got to trust my judgement when I say this woman shouldn’t have a biopsy done … they’ve got to cope with that sort of level of trust.”*

– medical officer (site 1).

Communication amongst the team was reported to require some extra effort but providers largely found this acceptable. Communication strategies differed across sites with two sites implementing assessment team meetings at scheduled times during the day with communication to coincide with discussion of reporting for several clients, and communication in between if needed. Another two sites used a less structured format where communication occurred client by client, via mobile phone or video-link. Importantly, each site used a communication strategy that suited team preferences and service workflows.

Strategies to ensure clear communication between remote and onsite providers included reducing background noise, speaking clearly and one person at a time, clarifying who was in a room during an assessment team meeting, spending time describing a client’s clinical presentation if expected to be important, and using marker arrows and description to talk about specific regions on images. At one site, a participant described their experience of communicating with the remote radiologist during synchronous telesonography. The participant took great care in talking to the remote radiologist through the ultrasound whilst being considerate of the client, who could only hear the participant’s side of the conversation (in that instance):

*“Oh maybe listening to my end of the conversation, makes people anxious. Yeah, and I do try and say, I try and remember to say to them ‘if I say something that you don’t understand, ask me because I don’t mean to say something that’s going to make you worried’.”*

*–* medical officer (site 4).

#### Adequate technical support and equipment

Initial setting up of technological aspects of the model were reported to be challenging at all sites. Existing telehealth networks within each jurisdiction were used to ensure secure transmission of client information and images. This involved considerable discussion and navigation through new processes for some providers involved in initial implementation. Compatibility of equipment, available bandwidth, age of equipment and fit-for-purpose communication equipment were other key technological considerations when implementing the remote radiology model.

The quality of imaging through synchronous telesonography required expert support from telehealth, medical physics and biomedical equipment experts to ensure that imaging transmitted using telehealth was diagnostically acceptable. For instance, the quality of transmitted images was dependent on the bandwidth available, which could cause some challenges for optimisation of imaging. The age of equipment was also reported to be an issue for transmission of quality imaging at one site. Testing of the quality of transmitted images was carried out in all jurisdictions, and issues were addressed by technical experts at the jurisdiction level in consultation with health professionals. In the absence of teleradiology specifications, there was a reliance on the remote radiologist to identify when the quality of imaging was not optimal during telesonography. Pixelated imaging was sometimes a concern at one site. This was reported by a remote radiologist to be frustrating and to slow the clinic, but was something that could be managed.

Issues with technology and equipment failing during clinics were sometimes a problem, but issues eased over time through increased experience using the technology. Participants reported that they adapted to use of the technology as time progressed by adding in specific processes such as testing the technology prior to a clinic to minimise failures and technical disturbances during scheduled remote radiology clinics. Service providers reported becoming ‘tech savvy’ as experience with the model grew, as described by one service manager (site 3): *“It took us a couple of goes … the first couple of clinics were a little bit hit and miss, but we improvised, and we made it work.”*

Having clinical service providers who were familiar with the technology and able to solve common technological issues enabled the smooth running of remote radiology assessment clinics. However, some participants commented on the lack of timely support for technological issues that occurred during clinics that were beyond the knowledge of service providers. Project officers engaged prior to, or in the early stages of, implementation provided a connection between onsite and remote providers, jurisdictional level management and technological experts, and also supported clinics with training and technical advice. A medical officer (site 4) commented that having a project officer available during the first few remote radiology assessment clinics was very useful “*because we … weren’t naturally attuned to the process*.”

## Discussion

The remote radiology assessment model for delivery of diagnostic assessment services for breast cancer is, we believe, the first of its kind reported in the literature. The model is an example of a successful telemedicine innovation in health service delivery that has emerged to address a need identified by local service providers and adapted to other Australian contexts. This qualitative exploration of the remote radiology assessment model from the perspective of service providers highlighted overall high satisfaction and important enablers and challenges related to implementation and ongoing delivery. These are significant to inform future implementation of such services.

This study found that a central foundation for successful implementation and operation of a safe telemedicine model was strong, collaborative relationships amongst service staff, and between service staff, key technology experts and governing bodies, with clear (but not overly restrictive) guidance from the overarching program management body. The remote radiology model was implemented with endorsement and support from the national body for quality management. This was achieved primarily through open lines of communication that allowed for the innovative idea to address local staffing issues to be voiced by local service staff, discussed with a jurisdictional governing body and then presented to the overarching management body. An organisational culture that is open to new ideas and willing to carry associated risks has been identified as important for supporting service innovation [26], and was an important factor in facilitating this innovation. The importance of involving relevant stakeholders for design and implementation of services involving teleradiology has been identified in other studies [27, 28].

As with implementation of any new model of service delivery, teamwork, trust and communication were central features for successful implementation and operation of the remote radiology model [10, 22]. With the involvement of a provider located remotely from the service staff and client, these features were particularly pertinent. There have been a number of studies which explored patient and provider communications for telehealth care delivery [29]. However the impact of telehealth on effective teamwork and communication between onsite and remote providers is less studied, but important given the growing use of telehealth models of care. For instance, videoconferencing may change how healthcare teams work together with potential for better communication and collaboration, and positive impacts on patient care [30].

The remote radiology model was operationalised in centres where a need for more timely access to services was observed by local staff. Implementation of the remote radiology model required considerable planning and was adapted for local staff characteristics, service flow preferences and equipment. Importantly, jurisdictions were able to apply the model within their own local operational environments while meeting the protocol requirements. Local responsiveness within the bounds of the guidelines is vital to enable customisation and implementation of an innovation that also operates safely [31–34]. Local innovation will continue as passionate service providers seek to provide high quality services for their populations, in the face of workforce challenges and inadequate service access, by exploiting technological advances and their application in telemedicine. Furthermore, these innovations can successfully address service accessibility and have potential to improve health equity [35–37]. Ensuring the local responsiveness or adaptability of innovations within guidelines is important for diffusion of novel approaches to health care delivery such as the remote radiology model.

Though local responsiveness is important in implementation, it has been established that, at a minimum, the safe use of teleradiology requires the use of available teleradiology guidelines, national and jurisdictional regulatory, monitoring and patient privacy processes, and a requirement for remote radiologists to meet professional standards and credentialing at the local site [16, 38, 39]. Furthermore, the use of teleradiology should be a supplement to, rather than a replacement for, existing services [16, 38, 39]. Currently, guidelines for the use of teleradiology for diagnostic procedures in breast assessment are lacking [40] and there is an absence of widely available guidelines for quality assurance of telesonography applications worldwide, to our knowledge. This study found that involvement of technology and equipment experts in preliminary discussions and throughout implementation was an essential component for the smooth, and safe implementation and operation of the model. This study also supports the view that continued technological support, training for users of the technology and fit-for-purpose equipment is crucial for a successful telemedicine model [30].

Notably, many of the factors for successful implementation of the remote radiology assessment model have been identified as constructs that influence implementation in the Consolidated Framework for Implementation Research [34]. The CFIR suggests constructs in five domains: characteristics of the intervention, inner and outer settings, individuals, and the implementation process [34]. Characteristics of the intervention that positively influenced implementation of the model included the ‘grassroots’ source of the model and the adaptability of the model for the local context of different clinics. Overlapping with these constructs, individual service providers who were passionate about equitable access to breast cancer diagnosis, and were appropriately skilled, were central in facilitating implementation of the model. The ongoing availability of experts once the model was operational was a positive inner setting construct, as were the robust networks and communication in each team. The guiding national protocol and existing teleradiology guidelines were outer setting constructs that had variable influences on implementation. Finally, engagement with experts in telehealth and telemedicine early in the development of the model was an important and enabling process construct for successful implementation of the model. Similarly, in the CFIR, the process of implementation is also assisted by reflection and evaluation, of which this project is arguably a component. Future evaluation and monitoring of innovative telemedicine models for service delivery could draw on the CFIR to inform assessment of contextual influences for effective implementation in different settings. This is particularly relevant in the field of telemedicine where the crossing of traditional service boundaries and the need for multiple sites for an intervention are inherent.

### Strengths and limitations

There was strong participation in interviews from a wide range of service providers using the remote radiology assessment model and providers involved in coordinating and supporting the model across jurisdictions. Sites had implemented the model with a staggered start and therefore experience in delivering the model varied across sites from one to 4 years at the time of data collection. Though methodologically unintended, this facilitated a more reliable longitudinal view of implementation and operation of the model, with views of early implementation (that were possibly more easily recollected by service providers in the sites with later starts), and views on later operation from service providers with more experience delivering the model, all able to be captured. Despite sites being located in three jurisdictions with some contextual differences, common factors for successful implementation and operation emerged strengthening the reliability and potential transferability of the findings. However, the findings came from just four sites in Australia, so caution needs to be applied in assuming transferability of the findings to other settings.

## Conclusion

The remote radiology assessment model, using telemedicine and technological advancements in radiological imaging, was innovated by service staff operating in a regional setting to overcome workforce challenges and provide a timely service to their clients. Understanding the key factors for supporting innovation, and implementing new and safe models of service delivery that incorporate telemedicine, will become increasingly important as technology evolves and becomes more accessible. It is possible to take proposed telemedicine solutions initiated by frontline workers and operationalise them safely and successfully: (i) through strong collaborative relationships that are inclusive of key experts; (ii) with clear guidance from overarching bodies with some flexibility for adapting to local contexts; (iii) through establishment of robust teamwork, trust and communication; and, (iv) with appropriate equipment and technical support.

## Supplementary Information

**Additional file 1.** Interview guide.

## Data Availability

The datasets generated and/or analysed during the current study are not publicly available for contractual and ethical reasons but may be available on reasonable request from the corresponding author. The approved project report may be available upon request from BreastScreen Australia.

## Abbreviations

BSANQMC: BreastScreen Australia National Quality Management Committee
PACS: Picture archiving and communication systems
RA: Remoteness Area
