# Supplementary material for: “From the technology came the idea”: safe implementation and operation of a high quality teleradiology model increasing access to timely breast cancer assessment services for women in rural Australia
Source: BMC Health Serv Res. 2020 Nov 30;20:1103. doi: 10.1186/s12913-020-05922-y (PMC7708244; doi:10.1186/s12913-020-05922-y)
Supplement: Supplementary file 1 — Additional file 1. Interview guide. [file 12913_2020_5922_MOESM1_ESM.docx]

Additional File 1. Interview guide

1. What is your current role in the remote radiology assessment clinics?
2. How long have you worked in remote radiology assessment clinics?
   1. Have you been involved in any other types of teleradiology? (providing or receiving advice)
3. Has your work changed since the remote radiology model has started here?
   1. If yes, how has your work changed?
4. Are there aspects of team functioning that have changed since implementation of the remote radiology assessment model?
5. Does the remote radiology assessment model affect how you access client information/records?
   1. If yes, in what way?

Probe, if required: could access to client data be improved?

Do you see any problems with access to client data in the current remote radiology model? (i.e. probe security concerns, sufficiency concerns)

1. In your view, are there any benefits of the remote radiology model for service staff?
   1. Probe further if required: How does this work?
   2. Do you think there are any negative effects associated with the remote radiology model for BreastScreen staff or service providers?
      1. Do you think that any of these negative effects could pose risks to staff or limit the service available?

Potential probe, if required: How does that work?

- 1. Check: what does this mean for service operation?

1. In your view, are there any benefits of the remote radiology model for clients?
   1. Probe further if required: How does this work?
   2. Do you think that any of these benefits specifically affect the quality and safety of care that clients receive?
   3. Do you think there are any negative effects associated with the remote radiology model for clients?
      1. Do you think that any of these negative effects influences the quality and safety of care that clients receive?

Potential probe, if required: How does that work?

- - 1. Standards for teleradiology are currently being developed, but could you comment on how you think the remote radiology model here conforms to existing standards that guide radiology?

Potential probe: PACS, image quality, environment

1. Thinking about the implementation or roll-out of the remote radiology model at this site, what worked well?
   1. Probe, if required: What specific processes worked well?
2. Were there any challenges to implementation of the model at this site?
   1. Probe, if required: what specific processes did not work well?
   2. Were the existing guidelines useful?
   3. What kind of support did you receive from organisations/units outside this clinic?
   4. Any opposition?
3. To what extent do you think service users/clients are aware of the new model of care?
   1. Do you think that the remote radiology model affects client satisfaction with this service?
      1. If yes: how so?
      2. If no: why not?
4. What could be done differently to improve the remote radiology assessment model at this site?
5. What advice would you provide to other sites seeking to implement the remote radiology assessment model?
6. Anything else you would like to add?
